# CRISPR-Mediated Knockout of the *ABCC2* Gene in *Ostrinia furnacalis* Confers High-Level Resistance to the *Bacillus thuringiensis* Cry1Fa Toxin

**DOI:** 10.3390/toxins12040246

**Published:** 2020-04-11

**Authors:** Xingliang Wang, Yanjun Xu, Jianlei Huang, Wenzhong Jin, Yihua Yang, Yidong Wu

**Affiliations:** College of Plant Protection, Nanjing Agricultural University, Nanjing 210095, China; wxl@njau.edu.cn (X.W.); 2016102098@njau.edu.cn (Y.X.); 2016202024@njau.edu.cn (J.H.); 2018102105@njau.edu.cn (W.J.); yhyang@njau.edu.cn (Y.Y.)

**Keywords:** Asian corn borer, *ABCC2*, CRISPR/Cas9, Cry1Fa, resistance

## Abstract

The adoption of transgenic crops expressing *Bacillus thuringiensis* (Bt) insecticidal crystalline (Cry) proteins has reduced insecticide application, increased yields, and contributed to food safety worldwide. However, the efficacy of transgenic Bt crops is put at risk by the adaptive resistance evolution of target pests. Previous studies indicate that resistance to *Bacillus thuringiensis* Cry1A and Cry1F toxins was genetically linked with mutations of ATP-binding cassette (ABC) transporter subfamily C gene *ABCC2* in at least seven lepidopteran insects. Several strains selected in the laboratory of the Asian corn borer, *Ostrinia furnacalis*, a destructive pest of corn in Asian Western Pacific countries, developed high levels of resistance to Cry1A and Cry1F toxins. The causality between the *O. furnacalis* *ABCC2* (*OfABCC2*) gene and resistance to Cry1A and Cry1F toxins remains unknown. Here, we successfully generated a homozygous strain (OfC2-KO) of *O. furnacalis* with an 8-bp deletion mutation of *ABCC2* by the CRISPR/Cas9 approach. The 8-bp deletion mutation results in a frame shift in the open reading frame of transcripts, which produced a predicted protein truncated in the TM4-TM5 loop region. The knockout strain OfC2-KO showed much more than a 300-fold resistance to Cry1Fa, and low levels of resistance to Cry1Ab and Cry1Ac (<10-fold), but no significant effects on the toxicities of Cry1Aa and two chemical insecticides (abamectin and chlorantraniliprole), compared to the background NJ-S strain. Furthermore, we found that the Cry1Fa resistance was autosomal, recessive, and significantly linked with the 8-bp deletion mutation of *OfABCC2* in the OfC2-KO strain. In conclusion, *in vivo* functional investigation demonstrates the causality of the *OfABCC2* truncating mutation with high-level resistance to the Cry1Fa toxin in *O. furnacalis*. Our results suggest that the *OfABCC2* protein might be a functional receptor for Cry1Fa and reinforces the association of this gene to the mode of action of the Cry1Fa toxin.

## 1. Introduction

Transgenic crops expressing *Bacillus thuringiensis* (Bt) insecticidal crystalline (Cry) proteins have been commercialized worldwide since 1996. The global planting area of Bt crops reached 104 million hectares in 2018 [1]. The widespread Bt crop adoption has suppressed pest populations, reduced insecticide usage, promoted biocontrol services, and economically benefited growers [2]. However, the efficacy of Bt crops is put at risk from the adaptive evolution of resistance by the target pests, and practical resistance to Bt crops has been documented at least in nine pest species in six countries [3,4,5].

The European corn borer *Ostrinia nubilalis* (Hübner) and the Asian corn borer *Ostrinia furnacalis* (Guenée) are two sibling species, both of which are economically important insect pests of corn, *Zea mays* (L.) [6]. *O. nubilalis* is present in Europe, North Africa, Central Asia, and North America [7], while *O. furnacalis* is distributed widely in East and Southeast Asia, Australia, and the Western Pacific Islands [8]. Bt corn expressing Cry1Ab has been widely planted for the control of some lepidopteran pests, including *O. nubilalis*, in North America since 1996, resulting in the suppression of target pest populations and reduced insecticide applications in both Bt and non-Bt corn [9,10]. No practical resistance to Cry1Ab has been identified in *O. nubilalis* field populations from North America for more than 20 years [5]. Bt corn expressing Cry1F has been used commercially in North America since 2003, and the frequency of alleles conferring Cry1F resistance did not increase in field populations of *O. nubilalis* sampled during 2003 to 2009 from the US corn belt [11]. However, practical resistance to Cry1F was discovered in 2018 from *O. nubilalis* populations from Nova Scotia of Canada [4]. It indicates that Bt resistane has already become a real threat to the long-term effectiveness of Bt corn for the control of *O. nubilalis*.

China is a major corn producer in the world and its corn acreage was 41.5 million hectares in 2018 [12]. *O. furnacalis* is the domiant pest and widely distributed in most of the corn-growing regions of China, while *O. nubilalis* is limited to some regions of northwestern China [13]. Although the commercial planting of Bt corn has not yet been approved in China, two Bt corn events (DBN9936 and Shuangkang12-5) obtained biosafety certificates in 2019 (MARA, 2020) [14], which is considered a prerequisite and landmark for commercial production. To be prepared for the switch to the adoption of Bt corn in the near future, a number of investigations have been conducted in China to assess resistance risk and cross-resistance by laboratory selection of *O. furnacalis* with Bt proteins. Under laboratory selection conditions, *O. furnacalis* developed high levels of resistance to various Cry1 toxins, including Cry1Ab, Cry1Ac, Cry1Ah, Cry1F, and Cry1Ie [15,16,17,18], proving its potential to develop Bt resistance in the field. Symmetrical cross-resistance was found among Cry1Ab, Cry1Ac, Cry1Ah, and Cry1F [15,16,17,18]. Asymmetrical cross-resistance was observed between Cry1Ie and other Cry1 toxins. Selection with Cry1Ab, Cry1Ac, Cry1Ah, or Cry1F did not confer cross-resistance to Cry1Ie, but selection with Cry1Ie resulted in high-level cross-resistance to Cry1F [15,16,17,18,19]. These results are valuable for the future designing of resistance management strategies for Bt corn in China. However, the resistance mechanisms underlying Bt resistance of *O. furnacalis* remain elusive. 

Several proteins have been identified and characterized as receptors for Cry toxins, including cadherins, aminopeptidase *N* (APN), alkaline phosphatases (ALP), and ATP-binding cassette (ABC) transporters [20]. One of the major mechanisms of resistance to Cry toxin is reduced toxin binding to their specific larval midgut receptors through the disruption of the receptor genes [21]. Since the disruption of the ABC transporter subfamily C2 (*ABCC2*) gene was first identified to confer Cry1Ac resistance in *Heliothis virescens* [22], mutations of the homologous *ABCC2* genes associated with Cry1A and/or Cry1F resistance have been found in several lepidopteran insects, including *Plutella xylostella*, *Trichoplusia ni* [23], *Bombyx mori* [24], *Helicoverpa armigera* [25], *Spodoptera exigua* [26], and *Spodoptera frugiperda* [27,28,29]. Recently, the CRISPR/Cas9 system has been applied to investigate the *in vivo* role of insect *ABCC2* in the mode of action and resistance mechanisms of Bt toxins. The causal relationship between *ABCC2* knockout and Cry1A/Cry1F resistance has been confirmed in *P. xylostella* [30], *S. frugiperda* [31], and *S. exigua* [32]. Interestingly, the knockout of either *ABCC2* or *ABCC3* of *H. armigera* did not confer Cry1Ac resistance, whereas the knockout of *ABCC2* and *ABCC3* together resulted in extremely high levels of resistance to Cry1Ac [33]. However, until now, whether or not the *ABCC2* gene of *O. furnacalis* (*OfABCC2*) is involved in Bt resistance development remains unknown.

In this study, we knocked out the *OfABCC2* employing the CRISPR/Cas9 system and constructed a homozygous mutant strain (OfC2-KO). Next, we performed toxicity bioassays and found that the *OfABCC2* knockout obtained a resistance to Cry1Fa greater than 300-fold compared to the wild-type control strain. Finally, we accessed the inheritance mode of the acquired resistance and confirmed the linkage between manipulated gene deletion and high-level resistance to Cry1Fa in the OfC2-KO strain.

## 2. Results

### 2.1. CRISPR/Cas9-Mediated Mutagenesis of OfABCC2 in O. Furnacalis

**Figure 1:**
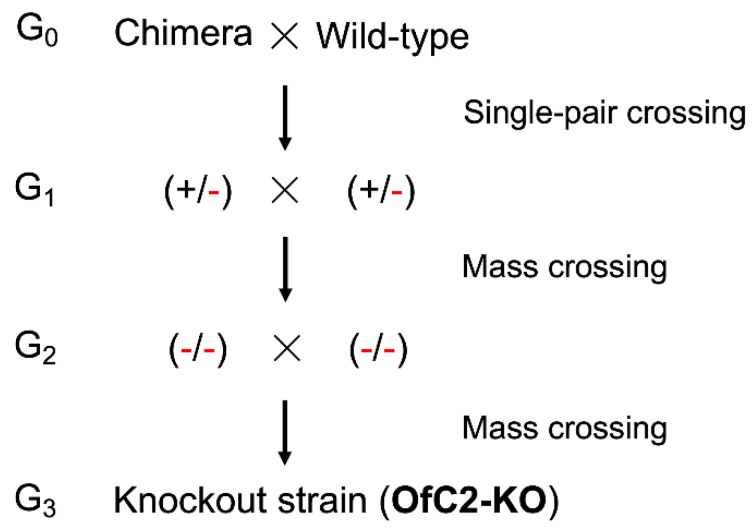
Diagram of the crossing strategy used to obtain the knockout strain homozygous for the 8-bp deletion mutation in exon 4 of *OfABCC2*. (+/-) means heterozygote (0/-8), (-/-) means mutant homozygote (-8/-8).

A total of 572 newly laid eggs (< 2 h) were injected with a mixture of the synthesized sgRNA and Cas9 protein. A total of 150 injected embryos (~26%) hatched and developed to 5th instar larvae. In order to obtain individuals with edited genomes as quickly as possible, the genomic DNA of 90 exuviates of the final instar larvae were isolated, and *OfABCC2* genotypes were identified by the direct sequencing of PCR products flanking the target site. Sequencing chromatograms revealed that 7.8% (7/90) of the examined G_0_ individuals were mutagenized at the target site with a stretch of double peaks. Then, the seven chimeras (six females and one male) were single crossed with the wild-type NJ-S moth, respectively (G_0_, Figure 1). 

After G_0_ oviposited, the genomic DNA of randomly selected eight–nine exuviates from each single-pair progeny were prepared, and their *OfABCC2* genotype was identified as described above. Among the 60 exuviates genotyped, 46 samples were wild-type homozygotes, seven individuals were heterozygotes harboring a wild-type allele and an 8-bp deletion allele, three samples were heterozygotes carrying a wild-type allele and a 1-bp insertion allele, and the genotype of the rest of the four individuals could not be identified by visual checks based on the sequencing chromatogram. We therefore confirmed efficient mutagenesis induced by CRISPR/Cas9 system had occurred in *OfABCC2* and the genome-edited alleles were transmitted to G_1_.

### 2.2. Construction of a Homozygous Strain with OfABCC2 Knocked Out

The mass mating was made among the above seven heterozygotes (three males and four females) that were harboring a wild-type allele and an 8-bp deletion allele (+/-) in G_1_ (Figure 1). The genomic DNA of 30 exuviates from G_2_ progeny were isolated and the genotype of *OfABCC2* was screened, and 21, five, and four samples were respectively identified as wild-type homozygotes (+/+), heterozygotes (+/-), and mutant homozygotes (-/-). The four moths (three females and one male) harboring both the 8-bp deletion alleles were mass crossed and their progeny (G_3_) was reared to form a homozygous knockout strain named OfC2-KO (Figure 1). Subsequently, the TA-clone sequencing of the PCR products using both gDNA and cDNA from the OfC2-KO larvae were performed, and confirmed the *OfABCC2* carrying the 8-bp deletion at the desired site (data not shown).

**Figure 2:**
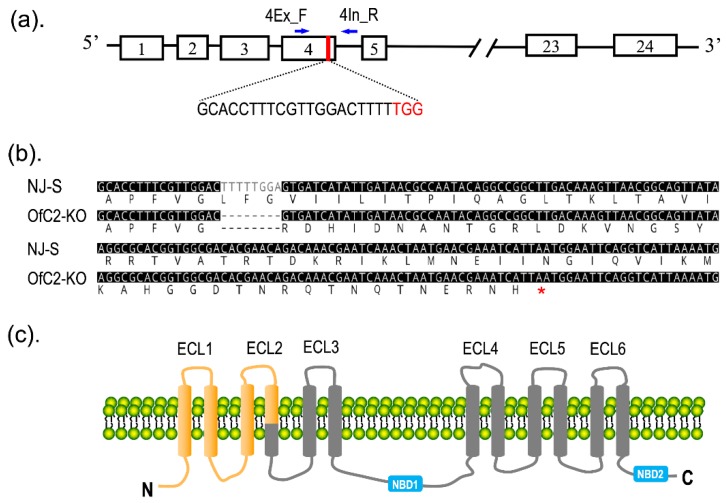
CRISPR/Cas9-mediated editing of the *OfABCC2* gene. (**a**) A diagram of the *OfABCC2* gene and sgRNA targeting site. The white boxes represent predicted exons through sequence alignment with *ABCC2*s from *Heliothis virescens* and *Plutella xylostella*. The sgRNA targeting site was located at exon 4, containing a proto spacer and a protospacer adjacent motif (PAM) sequence (TGG, in red). (**b**) The deduced peptide sequences from partial exon 4 to exon 6 of *OfABCC2*. The stop code is shown by a red asterisk. (**c**) A schematic diagram of the 12 transmembrane domains (TM1–TM12). The cleaved site induced by CRISPR/Cas9 is located at TM4, resulting in a frame shift of the transcript. The predicted protein produced from this mutant allele would be truncated in the intracellular TM4–TM5 loop of OfABCC2.

Based on the deduced peptide sequences, the 8-bp deletion at exon 4 caused a pre-mature stop codon (Figure 2a,b). The consequence of this 8-bp deletion is predicted to lose TM5-TM12 transmembrane segments and two nucleotide-binding domains (NBDs) (Figure 2c). In view of the absence of about 70% of the protein structure, the *ABCC2* gene in the OfC2-KO strain is predicted to be defective and most likely non-functional.

### 2.3. Impact of OfABCC2 Disruption on the Susceptibility of O. Furnacalis to Bt Toxins and Chemical Insecticides

Toxicity assays with larvae from the mutagenesis OfC2-KO strain and the background NJ-S strain against four Bt Cry toxins and two insecticides were carried out with the aim of assessing the impact of disrupted *OfABCC2* on larvae’s susceptibility. The bioassay results indicate that the OfC2-KO strain showed low levels of resistance to Cry1Ac (8.1-fold) and Cry1Ab (3.6-fold), but no significant resistance to Cry1Aa (1.4-fold) compared to the NJ-S strain based on LC_50_ values (Table 1). However, because the susceptibility of the OfC2-KO strain to Cry1Fa was reduced to a large extent, the LC_50_ cannot be obtained by bioassay. The mortality of the OfC2-KO larvae was only 4% when treated by 120 µg/g Cry1Fa, and the estimated resistance ratio was much more than 300-fold. In contrast, the two strains exhibited approximately equal susceptibility to two chemical insecticides (abamectin and chlorantraniliprole) with 0.6- and 1.3-fold difference of LC_50_s. Our findings provide strong reverse genetics evidence for *OfABCC2* involved in the toxicity and mode of action of Cry1Fa.

### 2.4. Dominance of Cry1Fa and Cry1Ac Resistance in the OfC2-KO Strain

To investigate the inheritance of different levels of resistance to Cry1Fa (high) and Cry1Ac (low) in the OfC2-KO strain, it was crossed with the susceptible NJ-S strain, and the responses of the two strains and their F_1_ progeny were determined at a diagnostic concentration of Cry1Fa (2 µg/g) and Cry1Ac (1 µg/g), respectively (Table 2). For Cry1Fa, the F_1a_ and F_1b_ progeny had a high mortality (100% and 98.3%) at 2 µg/g, and the dominance parameters (*h*) were 0 and 0.02. Similarly, for Cry1Ac, the corresponding mortality was 100%, and both of the two *h* values were 0. The results indicated that either a high level of resistance to Cry1Fa or a low level of resistance to Cry1Ac in OfC2-KO strain was inherited as a recessive mode.

### 2.5. Genetic Association between the 8-bp Deletion of OfABCC2 and Cry1Fa Resistance

**Figure 3:**
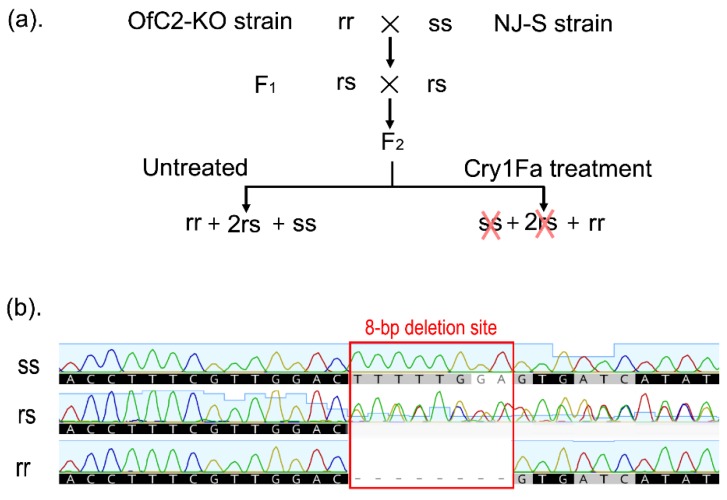
Linkage analysis of Cry1Fa resistance in the OfC2-KO strain of *O. furnacalis*. *OfABCC2* genotypes: *ss* = wild type; *rs* = heterozygous mutant; *rr* = homozygous mutant (8-bp deletion). (**a**) The crossing design used to generate F_2_ progeny (1*ss*: 2*rs*:1*rr*). (**b**) The direct sequencing chromatograms of PCR products amplified from a fragment of gDNA flanking the 8-bp deletion site (red box) of *OfABCC2*.

To clarify the causal relationship of the 8-bp deletion in exon 4 of *OfABCC2* with high levels of Cry1Fa resistance, a set of genetic crosses was performed (Figure 3a). By using direct-sequencing analysis of the target PCR products (see typical chromatogram in Figure 3b), the genotype of 25 individuals from NJ-S were homozygous for the wild-type (*ss*) and that of 30 individuals from the OfC2-KO strain were homozygous for the 8-bp deletion of *OfABCC2* (*rr*) (Table 3). When treated with 2 µg/g of Cry1Fa in F_2_ progeny, 22.6% (38/168) of the larvae survived after 7 days of treatment. All the detected 21 survivors randomly selected from the F_2_-treated group were homozygous for the 8-bp deletion of *OfABCC2* (*rr*), and the F_2_-untreated individuals were separated into wild-type homozygous (*ss*: 7), heterozygous (*rs*: *13*), and 8-bp deletion homozygous (*rr*: 9). Our results clearly demonstrated that the 8-bp deletion of *OfABCC2* is significantly linked (Fisher’s exact test, *p* < 0.0001) with Cry1Fa resistance in the manipulated OfC2-KO strain.

## 3. Discussion

In the current study, we successfully induced a deletion mutation of 8-bp into the *OfABCC2* gene of *O. furnacalis* by the CRISPR/Cas9 genome editing system, and characterized Bt resistance properties of the knockout OfC2-KO strain. We found that the OfC2-KO strain acquired a high level of resistance to Cry1Fa (>300-fold) and low levels of resistance to Cry1Ab and Cry1Ac (< 10-fold). We also confirmed the genetic association between the 8-bp deletion of *OfABCC2* and the obtained resistance to Cry1Fa in the knockout strain. These findings provide strong evidence that OfABCC2 plays a major role in meditating the toxicity of Cry1Fa in *O. furnacalis*. Moreover, the cross-resistance and inheritance pattern results provide helpful information for designing of resistance management strategies for future adoption of Bt corn in China. Furthermore, the OfC2-KO strain can be employed in an F_1_ screen program to investigate the diversity and frequency of the *OfABCC2* mutant alleles in field populations of *O. furnacalis*.

ABCC2 proteins have been identified as receptors for Bt toxins Cry1A and/or Cry1F in a number of lepidopteran insects, but they have differential contributions to the toxicities for individual Cry1 toxins. The CRISPR-mediated knockout of *P. xylostella ABCC2* conferred high levels of resistance to Cry1Aa, Cry1Ab, and Cry1Ac [30]. The double knockout of *ABCC2* and *ABCC3* of *H. armigera* confers a >15,000-fold resistance to Cry1Ac [33]. A point mutation in the *ABCC2* of *B. mori* resulted in high levels of resistance to Cry1Ab and Cry1Ac, but not to Cry1Aa [24]. The CRISPR-mediated knockout of *S. frugiperda ABCC2* conferred a 118-fold resistance to Cry1F [31], and the knockout of *S. exigua ABCC2* resulted in high levels of resistance to both Cry1Fa and Cry1Ac [32]. In our study, the knockout of *ABCC2* in *O. furnacalis* produced high-level resistance to Cry1Fa, low levels of resistance to Cry1Ab and Cry1Ac, and no resistance to Cry1Aa. The present study provides a new case for the investigation of the interaction between lepidopteran ABCC2 receptors and Bt Cry1 toxins.

A laboratory-selected strain of *O. nubilalis* developed a >1200-fold resistance to Cry1F, and the Cry1F resistance trait is controlled by a single quantitative trait locus (QTL) on linkage group 12 [34]. The subsequent fine mapping of the Cry1F resistance QTL identified a genomic region containing the *ABCC2* locus tightly linked with Cry1F resistance [35]. Practical resistance to Cry1F was recently documented in some field populations of Canadian *O. nubilalis* [4]. It will be interesting to check whether there are mutations in the *ABCC2* gene in both the laboratory-selected strain and field-derived resistant populations of *O. nubilalis*. The identification of the specific gene for Cry1F resistance of *O. nubilalis* is urgently needed for developing molecular tools to monitor the spreading of the practical resistance in the field.

Several studies reported the potential mechanisms of Cry1Ab and Cry1Ac resistance in the laboratory-selected strains of *O. furnacalis*, such as the up-regulation of the V-ATPase catalytic subunit A and heat shock 70 kDa proteins [36], the down-regulation and mutation of a cadherin gene [37], the differential expression of the miRNAs targeting potential Bt receptors [38], and the reduced expression of APN and ABC subfamily G transcripts [39]. The CRISPR-mediated knockout approach established for *O. furnacalis* in the present study can be used to evaluate the functional role of the candidate genes relating to Bt resistance.

The characterization of the inheritance of Bt resistance will provide important information for evaluating the risks of evolution of resistance and will make it possible to formulate effective resistance management strategies. Based on previous reports, resistance to Cry1-type toxins mediated by *ABCC2* mutations was recessive or incompletely recessive [22,23,24,27,28,30,32,33]. Consistent with these results, both the high-level resistance to Cry1Fa (>300-fold) and low-level resistance to Cry1Ac (~8-fold) were inherited as a recessive mode in the knockout OfC2-KO strain of *O. furnacalis*.

In the present work, the obtained Cry1Fa resistance was confirmed to be genetically associated with the 8-bp deletion of *OfABCC2*, which excludes the CRISPR-mediated off-target effects on resistance phenotype. We analyzed 18 research cases that employed the CRISPR/Cas9 system to manipulate the resistance genes to Bt toxins or insecticides, and found that only five of them performed linkage analysis between acquired resistance and the introduced mutation, including the knockout of the cadherin gene in *H. armigera* [40], nicotinic acetylcholine receptor α6 subunit in *P. xylostella* and *S. exigua* [41,42], the ryanodine receptor G4946E mutation in *Drosophila melanogaster* [43], and a *CYP9M10* gene in *Culex quinquefasciatus* [44]. We therefore recommend that when CRISPR-based gene editing is conducted to verify the function of a candidate gene, it is necessary to perform a genetic linkage analysis in order to clarify whether there are off-target effects.

## 4. Materials and Methods 

### 4.1. Insect Strains and Rearing

The susceptible NJ-S strain was originally collected from Nanjing, China, in May 2010, and has been maintained in the laboratory without exposure to any insecticides or Bt toxins. By using the CRISPR/Cas9 genome-editing system, the *OfABCC2* gene in the background strain NJ-S was knocked out to construct a manipulated strain denoted as OfC2-KO. The genome-edited OfC2-KO strain is homozygous for the 8-bp deletion in exon 4 of *OfABCC2*, which was predicted to produce a truncated and loss-of-function protein.

The larvae of *O. furnacalis* were reared on an artificial diet with corn and soybean powder as major ingredients at 27 ± 1 °C, 80% relative humidity (RH), and a photoperiod of 16 h light:8 h dark. The pupae were transferred to mating cages with more than 80% RH and a photoperiod of 16:8 h (L: D). Adults were supplied with 10% sugar solution to replenish energy. About 5–6 pieces of waxed papers, as substrate for oviposition, were placed on the top of the cage, and the bottom sheet was collected daily. Egg masses were incubated in plastic boxes lined with moistened filter paper until hatching.

### 4.2. Diet Bioassay

The activated Cry1Aa, Cry1Ab, Cry1Ac, and Cry1Fa toxins were purchased from Marianne Pusztai-Carey (Case Western Reserve University, Cleveland, OH, USA). Abamectin (20 g/L EC) was obtained from the Institute of Plant Protection, Guangdong Academy of Agricultural Sciences, Guangzhou, Guangdong Province, China. Chlorantraniliprole (200 g/L SC) was purchased from DuPont Agricultural Chemicals Ltd. (Wilmington, DE, USA).

We used the diet incorporation method to evaluate the toxicity of Cry toxin or chemical insecticide to *O. furnacalis*. Briefly, 5 to 7 concentrations of Bt or insecticide test solutions were first diluted in distilled water. Then, we added the solution (or distilled water for control) to a proper amount diet in a clean mixing bowl and thoroughly mixed all the ingredients together until a soft, smooth dough was obtained. Next, we dispensed the toxin-incorporated diet into each well of a 24-well plate. After the diet cooled and solidified, one *O. furnacalis* larva (neonate for Cry toxin susceptibility test and 2nd instar larva for chemical insecticide bioassay) was placed in each well. All the plates were kept at an illumination incubator set at 27 ± 1 °C, 80% RH, and a photoperiod of 16:8 h (L:D). For Cry toxin, the mortality was recorded after 7 days of treatment, and the larvae were considered dead if they died or weighed less than 5 mg. For abamectin and chlorantraniliprole, the mortality was recorded after 2 days of treatment, and the larvae were considered dead if they did not move after gentle prodding with a brush. The data were analyzed with PoloPlus (LeOra Software) [45] to estimate the LC_50_ with 95% fiducial limits (FL), as well as the slopes of the concentration–mortality lines. Resistance ratios (RR) were calculated by dividing the LC_50_ for a particular strain by the LC_50_ for the susceptible NJ-S strain.

### 4.3. Design and Preparation of sgRNA

In our previous work, the full-length sequences of *OfABCC2* mRNA (GenBank no. MN783372) had been obtained from the susceptible NJ-S strain of *O. furnacalis*. By scanning the GN_19_NGG motifs, we identified a sgRNA target site (5′-GCACCTTTCGTTGGACTTTTTGG-3′) in predicted exon 4 of *OfABCC2* (Figure 2a). A PCR-based approach was employed to prepare sgRNA according to the instructions [46]. In brief, a forward oligonucleotide encoding a T7 polymerase-binding site and the sgRNA target sequences GN_19_ (OfC2_sgF, Table 4) and a universal oligonucleotide encoding the remaining sgRNA sequences (OfC2_sgR, Table 4) were designed at first. The OfC2_sgF and OfC2_sgR were fused by PCR to generate a sgRNA DNA template. The PCR reaction mixture (50 μL) contained 10 μL of 5 × PCR buffer, 4 μL of 2.5 mM dNTP, 4 μL of 10 μM OfC2_sgF, 4 μL of 10 μM OfC2_sgR, 0.5 μL of PrimeSTAR polymerase (TaKaRa, Dalian, China), and 27.5 μL of Nuclease-free water. PCR was performed at 98 ℃ for 30 s, 30 cycles (98 ℃ 5 s, 60 ℃ 30 s, 72 ℃ 15 s), 72 ℃ for 10 min, and holding at 4 ℃. After identification by electrophoresis, the PCR products were purified by a QIAprep^®^ Spin Miniprep Kit (QIAGEN, Hilden, Germany). A MEGAshortscript™ T7 High Yield Transcription Kit (Ambion, Foster City, CA, USA) was used for sgRNA in vitro transcription according to the manufacturer’s protocol.

### 4.4. Egg Collection and Microinjection

Mated female moths of *O. furnacalis* were allowed to lay egg masses on pieces of wax paper previously placed on the top of the mating cage. Fresh egg masses (within 2 h after oviposition) were immediately collected by cutting the wax paper. Then, the eggs were lined on double-sided adhesive tape on a microscope slide. About 1 nL of mixture containing 150 ng/μL of Cas9 protein (GeneArt™ Platinum™ Cas9 Nuclease, Thermo Fisher Scientific, Shanghai, China) and 300 ng/μL of sgRNA were injected into each egg using a FemtoJet and InjectMan NI 2 microinjection system (Eppendorf, Hamburg, Germany). After injection, the embryos were incubated in an incubator as described above. The hatched larvae were fed an artificial diet without any toxin.

### 4.5. Generation of OfABCC2 Mutation Mediated by CRISPR/Cas9

After embryo injection, the genomic DNAs of exuviate from 90 5th-instar larva were isolated individually using an AxyPrep Multisource Genomic DNA Miniprep Kit (Axygen, Hangzhou, China) following the manufacturer’s instruction. To identify the indel mutations at predicted exon 4 of *OfABCC2*, the intron 4 sequences was first amplified by a specific pair of primers (4Ex_F and 5Ex_R, Table 4) and then by using the primers of 4Ex_F and 4In_R (Table 4) to amplify a 280-bp region flanking the desired cleavage site. The second PCR reaction mixture contained 1 μL of template, 1 μL of each of the 4Ex_F or 4In_R primer, 12.5 μL of 2× Taq Master Mix (TaKaRa, Dalian, China), and 9.5 μL of PCR-grade water in a final volume of 25 μL. PCR was performed at 95 ℃ 3 min, 35 cycles (95 ℃ 30 s, 55℃ 30 s, 72 ℃ 1 min), 72 ℃ for 10 min, and 4 ℃ forever, and then the PCR products were directly sequenced with 4Ex_F (sequencing primer) by TSINGKE Biological Technology (Nanjing, China). Direct sequencing chromatograms of mutant *OfABCC2* have double peaks around the cutting site at G_0_ generation. To detect the detailed deletion information of G_2_ genomic DNAs, the 280-bp PCR products were subcloned into pTOPO-T vector (Aidlab Biotechnologies, Beijing, China) and sequenced by TSINGKE Biological Technology. The acquired 8-bp deletion in *OfABCC2* was reconfirmed by clone sequencing using genomic DNA and mRNA templates from the knockout strain OfC2-KO.

### 4.6. Inheritance Model Determination and Genetic Association Analysis

The sex of *O. furnacalis* was visually determined based on the bottom characters of the pupa. Male adults (30 moths) from the original strain NJ-S were mass crossed with virgin female adults (30 moths) of the knockout strain OfC2-KO and vice versa. The degree of dominance (*h*) was estimated using the formula: *h* = (S*rs* − S*ss*)/(S*rr* − S*ss*), where S*rs*, S*ss*, and S*rr* are the survival rate for F_1_ hybrids, the NJ-S strain, and the OfC2-KO strains, respectively. The *h* varies from 0 for completely recessive resistance to 1 for completely dominant resistance [47].

For genetic association analysis between the 8-bp deletion of *OfABCC2* and Cry1Fa resistance phenotype, the F_1_ progeny from the reciprocal crosses were pooled and mass crossed to produce F_2_ progeny (Figure 3a). A total of 168 newly hatched larvae of the F_2_ progeny were treated with 2 μg/g of Cry1Fa toxin. The survivors (F_2_-treated) were collected after 7 days of treatment. The DNA from random selected parents (NJ-S and OfC2-KO), F_2_-treated survivors, and F_2_-untreated individuals were extracted for *OfABCC2* genotyping.

## Figures and Tables

**Table 1:** Toxicity response to four Bt toxins and two chemical insecticides of larvae from the original NJ-S and OfC2-KO strains of *O. furnacalis*.

Toxin/Insecticide	Strain	N ^1^	Slope ± SE	LC_50_ (μg/g)	95% Fiducial Limits	RR ^2^
Cry1Aa	NJ-S	312	3.714 ± 0.519	0.391	0.320-0.455	1 1.4
	OfC2-KO	384	2.583 ± 0.386	0.527	0.359-0.737	
Cry1Ab	NJ-S	360	2.978 ± 0.362	0.116	0.074-0.177	1 3.6
	OfC2-KO	384	2.339 ± 0.286	0.414	0.259-0.585	
Cry1Ac	NJ-S	720	3.248 ± 0.427	0.100	0.069-0.136	1 8.1
	OfC2-KO	384	3.531 ± 0.572	0.808	0.676-0.947	
Cry1Fa ^3^	NJ-S	408	4.488 ± 0.505	0.411	0.349-0.466	1 >300
	OfC2-KO	48	-	-	-	
Abamectin	NJ-S	192	2.221 ± 0.227	0.118	0.090-0.153	1 1.3
	OfC2-KO	432	1.937 ± 0.171	0.153	0.122-0.188	
Chlorantraniliprole	NJ-S	432	2.106 ± 0.217	0.031	0.025-0.037	1 0.6
	OfC2-KO	432	1.387 ± 0.137	0.018	0.013-0.023	

^1^ Numbers of larvae used in bioassay; ^2^ RR (resistance ratio) = LC_50_ (OfC2-KO)/LC_50_ (NJ-S); ^3^ LC_50_ for OfC2-KO could not be determined because of an insufficient dose response (only 4% mortality at 120 μg/g of Cry1Fa treatment).

**Table 2:** Mortality and dominance of the susceptible NJ-S strain, OfC2-KO strain, and their F_1_ progeny from reciprocal crosses to the diagnostic concentration of Cry1Fa and Cry1Ac, respectively.

Strain/cross	Treatment	N ^1^	Survival Number	*h* ^2^
NJ-S	Cry1Fa	72	0	
	Cry1Ac	48	0	
OfC2-KO	Cry1Fa	72	67	
	Cry1Ac	96	37	
F_1a_ (OfC2-KO♀×NJ-S♂)	Cry1Fa	120	0	0
	Cry1Ac	120	0	0
F_1b_ (OfC2-KO♂×NJ-S♀)	Cry1Fa	120	2	0.02
	Cry1Ac	120	0	0

^1^ Numbers of larvae measured at the Cry1Fa (2 μg/g) or Cry1Ac (1 μg/g) diagnostic concentration; ^2^ The degree of dominance (*h*) = (survival of F_1_ - survival of NJ-S)/(survival of OfC2-KO - survival of NJ-S). *h* = 0, completely recessive; *h* = 1, completely dominant.

**Table 3:** Genetic linkage between the 8-bp deletion of *OfABCC2* and resistance to Cry1Fa in *O. furnacalis*.

F_2_ Progeny ^1^	Number of Individuals for Each Genotype ^2^		
	*ss*	*rs*	*rr*
NJ-S	25	0	0
OfC2-KO	0	0	30
F_2_-untreated larvae (n = 29)	7	13	9
F_2_-treated survivors (n = 21)	0	0	21

^1^ F_1_ progeny between the susceptible NJ-S and Cry1Fa-resistant *OfABCC2* strains were crossed to produce F_2_ progeny. 168 larvae from the F_2_ progeny were treated with 2 μg/g of Cry1Fa toxin. 21 of 38 survivors and 30 untreated larvae were genotyped individually by direct sequencing of the PCR products; ^2^ *ss* represent homozygous for the wild type *OfABCC2*, while *rs* means heterozygous for the 8-bp deletion allele of *OfABCC2*, and *rr* represent homozygous for the 8-bp deletion allele of *OfABCC2*.

**Table 4:** Primers used in this study.

Primer Name	Primer Sequences (5′ to 3′)
OfC2_sgF	GAAATTAATACGACTCACTATAGCACCTTTCGTTGGACTTTTGTTTTAGAGCTAGAAATAGC
OfC2_sgR	AAAAGCACCGACTCGGTGCCACTTTTTCAAGTTGATAACGGACTAGCCTTATTTTAACTTGCTATTTCTAGCTCTAAAAC
4Ex_F	TAAACCAAGTGTCCATAGGAGACG
5Ex_R	TTCGTTTGTCTGTTCGTGTCGC
4In_R	GCTGACTATGACATCCACAAAGACAA
